# Reliance on Clinical Signs and Symptoms Assessment Leads to Misuse of Antimicrobials: *Post hoc* Analysis of 350 Chronic Wounds

**DOI:** 10.1089/wound.2021.0146

**Published:** 2022-09-15

**Authors:** Thomas E. Serena, Lisa Gould, Karen Ousey, Robert S. Kirsner

**Affiliations:** ^1^ SerenaGroup® Research Foundation, Cambridge, Massachusetts, USA.; ^2^ South Shore Health Department of Surgery (or Brown Alpert Department of Medicine), Weymouth, Massachusetts, USA.; ^3^ School of Human and Health Sciences, University of Huddersfield, West Yorkshire, United Kingdom.; ^4^ Dr. Phillip Frost Department of Dermatology and Cutaneous Surgery, University of Miami Miller School of Medicine, Miami, Florida, USA.

**Keywords:** antimicrobial stewardship, antibiotic prescribing, bacterial burden, chronic wounds, clinical decision support, diagnostic pathway, wound clinic

## Abstract

**Objectives:**

Bacteria frequently impede wound healing and cause infection. Clinicians rely on clinical signs and symptoms (CSS) to assess for bacteria at the point of care, and inform prescription of antibiotics and other antimicrobials. Yet, robust evidence suggests that CSS has poor sensitivity for detection of problematic bacterial burden and infection, hindering antimicrobial stewardship efforts. This study evaluated CSS-based antimicrobial prescribing practices across 14 wound care centers.

**Approach:**

Data were analyzed from the fluorescence assessment and guidance (FLAAG) trial, a study of 350 chronic wounds across 20 clinicians. Clinicians reviewed patient history and assessed for CSS using the International Wound Infection Institute infection checklist. Wounds with >3 criteria or any overwhelming symptom were considered CSS+. Bacterial levels were confirmed with quantitative tissue culture of wound biopsies.

**Results:**

Antimicrobials (including dressings, topicals, and systemic antibiotics) were prescribed at a similar rate for wounds identified as CSS+ (75.0%) and CSS− (72.8%, *p* = 0.76). Antimicrobial dressings, the most frequently prescribed antimicrobial, were prescribed at a similar rate for CSS+ (83.3%) and CSS− (89.5%, *p* = 0.27) wounds. In 33.3% of patients prescribed systemic antibiotics, no CSS were present. Prescribing patterns did not correlate with bacterial load.

**Innovation:**

This study is the first to evaluate antimicrobial prescribing trends in a large, multisite cohort of chronic wound patients.

**Conclusions:**

Reliance on CSS to diagnose clinically significant bacterial burden in chronic wounds leads to the haphazard use of antimicrobials. Improved methods of identifying bacterial burden and infection are needed to enhance antimicrobial stewardship efforts in wound care. Clinicaltrials.gov ID. NCT03540004.

**Figure:**
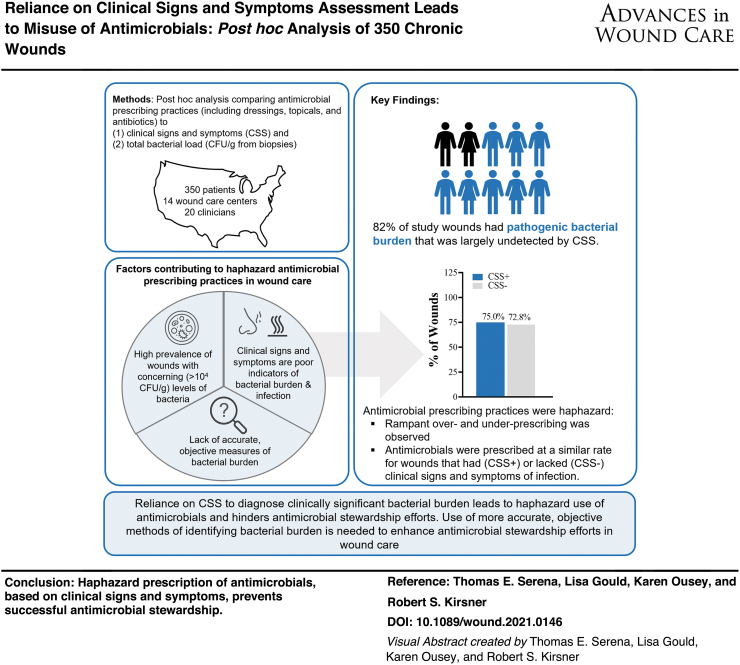


**Figure:**
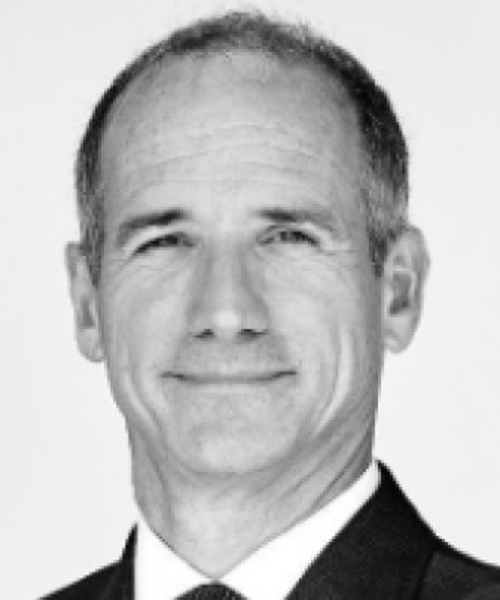
Thomas E. Serena, MD

## Introduction

Despite the availability of advanced therapies, less than half of chronic wounds achieve closure within 12 weeks.^1^ Patients with chronic wounds experience a decreased quality of life, loss of function and in some cases, significant morbidity.^2^ The accumulation of bacteria at concentrations >10^4^ colony-forming units per gram of tissue (CFU/g) has been associated with an increased risk of delayed wound healing.^3–5^ In a 228-patient randomized controlled trial, presence of bacterial loads >10^4^ CFU/g had a significant negative effect on healing.^3^ Similarly, others have shown delayed healing when bacterial loads >10^5^ CFU/g are present.^4,6^ Therefore, reducing this clinically significant level of bacterial burden is a rational approach to improve healing.

Wound clinicians must work efficiently to identify and appropriately reduce bioburden in wounds to facilitate healing and treat or prevent infection. Treatment methods employed to reduce bioburden and facilitate wound healing vary depending on the type and size of wound, patient history, and other comorbidities, but typically include debridement, pressure off-loading, application of appropriate dressings, and increasingly the use of antimicrobials.^7,8^

Antimicrobials, which include antiseptics as well as antimicrobial dressings (*e.g.*, silver impregnated dressings), topical, oral and intravenous antibiotics, are a routine part of management of bacterial burden; however, across specialties up to 50% of antibiotic prescriptions are unnecessary or inappropriate.^9^

Chronic wound patients receive significantly more antibiotic prescriptions than age- and gender-matched patients without wounds.^10^ Physicians concerned with failing to detect infection in a timely manner may prescribe antimicrobial agents in the absence of evidence supporting critical levels of bacteria. Analysis of antibiotic prescribing behaviors in the United Kingdom between 2013 and 2015 revealed that wounds accounted for 16.5% of all systemic antibiotic prescriptions.^11^

Clinicians are often pressured by patients to prescribe antibiotics as they are viewed as the safe option.^12^ However, unlike most drug therapies, antibiotics become less effective with use; chronic wounds including diabetic foot, venous, arterial and pressure ulcers, often harbor multiple drug-resistant bacteria, and form biofilms blocking access to antibiotic and topical antimicrobial access.^13^

### Innovation

Reliance on clinical signs and symptoms (CSS) to diagnose levels of bacteria that may impede healing of chronic wounds resulted in both under- and overprescribing of antimicrobials; this trend was consistent across multiple wound types and outpatient wound care centers. Our results highlight how empirical prescription of antimicrobials routinely misses the mark, hindering antimicrobial stewardship efforts. The field of wound care must therefore work toward expanding education, additional research on the effectiveness of antiseptics, antimicrobials, and antibiotics in treating wound infections, and adoption of techniques and technologies to aid in diagnosis of elevated bacteria levels.

### Clinical problem addressed

In standard care, clinicians routinely rely on clinical evaluation of CSS of infection to inform selection of antimicrobials at the point of care. Unfortunately, given the lack of standardized training across providers, detection of CSS is inconsistent and resulting diagnosis and treatment may be heterogeneous at best, and potentially seriously flawed. Even if correctly detected, several studies report that CSS lack sensitivity in detecting elevated levels of bacteria indicative of delayed healing or infection.^14–16^

In a large, 20-clinician trial, sensitivity of CSS to detect bacterial loads >10^4^ CFU/g was <15% across multiple wound types, resulting in >80% of wounds with bacterial loads >10^4^ CFU/g being missed.^14^ Similar findings were reported by Gardner *et al.*, who observed high bacterial loads (>10^6^ CFU/g) in 39% of DFUs, yet found that no individual sign included in the Infectious Disease Society of America criteria for infection was able to predict which wounds had these high bacterial loads.^17^

Indeed, patients with chronic wounds, particularly diabetic foot ulcers (DFUs), may not exhibit the typical CSS indicative of infection.^18,19^ These inconsistencies in detecting infection-causing bacteria combined with the failure to mount CSS in the presence of certain comorbidities (*i.e.,* diabetes, autoimmune disease) may lead to widespread antibiotic misuse in wound care.^12^

In 2015, the World Health Organization^20^ outlined and endorsed a global action plan to tackle antimicrobial resistance, highlighting strategic objectives, including improving awareness and understanding of antimicrobial resistance; strengthening knowledge through surveillance and research; reducing the incidence of infection; optimizing antimicrobial use. The Joint Commission (a global quality improvement organization that accredits and certifies hospitals in the United States) mandates that all outpatient departments that prescribe antimicrobials have an antimicrobial stewardship plan (ASP) in place.^21^

Since infection is one of the most common complications encountered in wound care, it is critical to ensure that wound care providers understand the scope of the antimicrobial prescribing problem and are educated on appropriate use of antimicrobials to manage bacterial burden. Obtaining baseline data on the patterns of antimicrobial prescribing in the outpatient wound clinic is an essential step in developing an ASP.^22^ This study examined how CSS informed antimicrobial prescribing across 14 outpatient wound care centers.

## Materials and Methods

### Study population and design

*Post hoc* analysis was performed on the data from the Fluorescence Assessment and Guidance (FLAAG) clinical trial conducted in 2018.^23^ This prospective, single-blind, multicenter cross-sectional clinical trial (Clinicaltrials.gov #NCT03540004) was conducted across 14 outpatient wound care centers in the United States and included 20 experienced wound care specialists (12 podiatrists, 1 emergency room physician, 5 wound care physicians, and 2 nurse practitioners). The study included adult (>18 years old) patients presenting with wounds of unknown infection status, including diabetic foot, venous leg ulcers, pressure ulcers, surgical wounds, and others.

At least 20 subjects were represented in each major chronic wound type. Broad inclusion and minimal exclusion criteria ensured a fair representation of “real-world” wounds in this trial, as previously described.^23^ The study received ethics approval by a central institutional review board (Veritas IRB, Montreal, Canada). The FLAAG trial sponsor provided permission to study coauthors to access anonymized data reporting outcomes of the clinical assessment and 4-week treatment plan for the purpose of this *post hoc* analysis.

### Data collection

Clinical investigators performed a history and physical examination that included assessment of wounds for CSS using International Wound Infection Institute (IWII) Wound Infection checklist criteria. The checklist includes both overt and covert criteria, which can occur simultaneously in a wound^24^ (Table 1). Wounds with ≥3 criteria in any one category, or one or more overwhelming sign or symptom (*e.g.,* significant amount of purulent discharge), were considered positive for CSS (CSS+).

**Table 1.** *Signs and symptoms of infection based on the International Wound Infection Institute checklist* ^24^

Local Infection				Spreading Infection	
*Covert (Subtle Signs)*		*Overt (Classic) Signs*		Extending induration ± erythema Lymphangitis Crepitus Wound breakdown/dehiscence with or without satellite lesions Malaise/lethargy or nonspecific general deterioration Loss of appetite Inflammation, swelling of lymph glands	
Hypergranulation (excessive “vascular” tissue) Bleeding, friable granulation Epithelial bridging and pocketing in granulation tissue Wound breakdown and enlargement Delayed wound healing beyond expectations New or increased pain Increasing malodor		Erythema Local warmth Swelling Purulent discharge Delayed wound healing beyond expectations New or increasing pain Increased malodor			
Number of covert signs present: 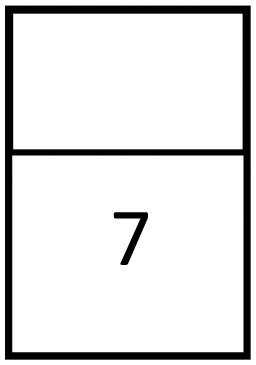		Number of spreading signs present: 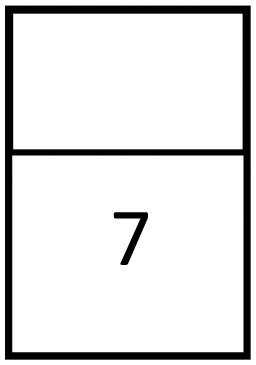		Number of spreading signs present: 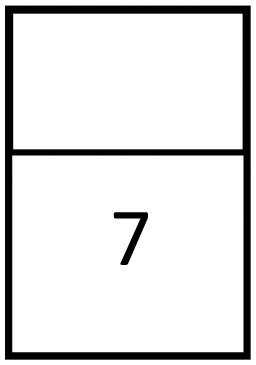	

Based on CSS assessment, the investigators recorded a 4-week treatment plan for the wound, including the use of antimicrobial dressings, or topical antimicrobial and/or topical or systemic antibiotics. A biopsy of the wound (6 mm in diameter and 2 mm in depth) was obtained and processed by a single, accredited, third-party laboratory for quantitative analysis of total bacteria load (Eurofins Central Laboratory, Lancaster, PA) as described in a previous study^25^; the laboratory was blinded to wound CSS status.

These procedures were part of the larger FLAAG trial, which also included capture of fluorescence images of the wound to detect elevated bacterial burden and creation of a revised treatment plan based on this information.^23^ The data described here solely represent the CSS assessment and associated treatment planning, with no consideration of the fluorescence imaging results. An electronic laboratory notebook was not used.

### Statistical analysis

For the purposes of analysis, the term “antimicrobials” includes antimicrobial bandages, topical antimicrobials, and antibiotics (topical, oral, or intravenous). Categorical data were analyzed using chi-square tests, and nonparametric continuous data were analyzed using the Mann–Whitney U test or the Kruskal–Wallis H test, as appropriate.

Correlations between continuous variables were evaluated using Spearman's coefficient of rank correlation (rho). Logistic regressions were conducted to evaluate the demographic factors and types of CSS that may contribute to antimicrobial prescribing (including topical and systemic antibiotics) using stepwise entry; variables were entered into the model if *p* < 0.2 and removed if *p* > 0.5. False discovery rate (FDR) was determined to correct for multiple comparisons with adjusted *p*-values (*q*-values) reported for significant findings (*p*-value <0.05).

## Results

A total of 371 patients with various wound types were assessed for CSS of infection based on the IWII criteria, and microbiology data were completed for 350 patients. Twenty-one patients with incomplete microbiology data were not included in the analysis. The final data set included the following: DFUs (*n* = 138), pressure ulcers (*n* = 22), surgical wounds (*n* = 60), venous leg ulcers (*n* = 106), and other wounds (*n* = 24).

Based on clinical assessment of IWII criteria (Table 1), investigators identified 86% of wounds (302/350) as negative for CSS (CSS−), while 14% (48/350) of study wounds were identified as positive for CSS (CSS+). The IWII criteria classify clinical signs and systems into three divisions of the wound infection continuum: covert infection, overt infection, or spreading infection. Most CSS+ wounds had ≥3 covert infection criteria (81.3% of CSS+ wounds), while 56.3% of CSS+ wounds had ≥3 overt infection criteria; only 10.4% of CSS+ wounds had ≥3 signs and symptoms of spreading infection.

**Figure 1.**
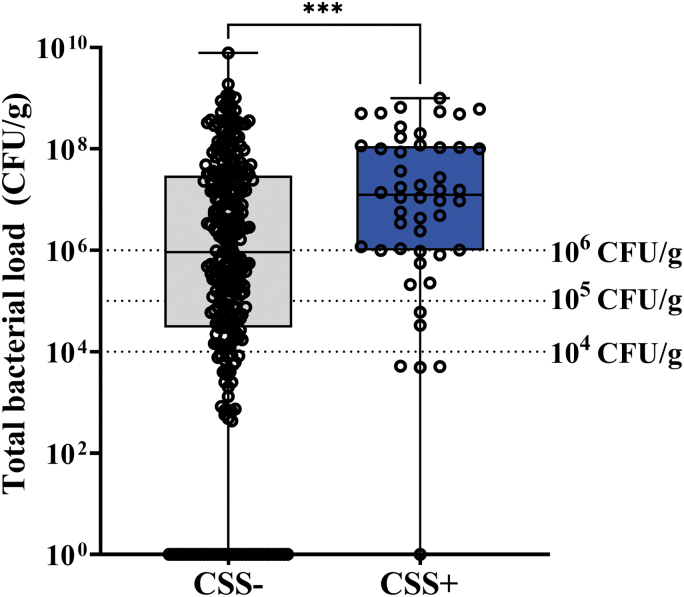
Total bacterial load of study wounds identified as negative (CSS−) or positive (CSS+) for CSS based on International Wound Infection Institute (IWII) wound infection criteria. Box and whisker plot of total bacterial load for wounds deemed CSS− (*n* = 302) and CSS+ (*n* = 48). *Open circles* represent individual study wounds; *middle lines* indicate median bacterial load; error bars indicate range. Of the CSS− wounds, 36 had total bacterial load of 0. *Dashed lines* at 10^4^ CFU/g represent minimum bacterial threshold at which delayed healing is observed; *dashed lines* at 10^5^ CFU/g and 10^6^ CFU/g represent minimum bacterial thresholds at which wounds are considered infected and treatment is warranted. ***p < 0.001 by the Mann–Whitney test of log-transformed data. CSS, clinical signs and symptoms; CFU, colony forming units.

Bacterial loads >10^4^ CFU/g were observed in 82% (287/350) of study wounds, while 52.2% of study wounds had bacterial loads >10^6^ CFU/g, a level some consider to be indicative of infection (Fig. 1).^16,26^ CSS criteria had poor sensitivity (<15%) for identifying wounds with high bacterial burden. For additional details on the diagnostic accuracy of CSS in the FLAAG trial, readers are directed to the publication of the primary endpoints of the trial reported by Le *et al.*^23^

Most CSS− wounds had total bacterial load (TBL) >10^4^ CFU/g (80.2%), and almost half of CSS− wounds (48.6%) had bacterial loads >10^6^ CFU/g; only 19.8% of wounds deemed CSS− had bacterial loads <10^4^ CFU/g. Median TBL of CSS− wounds was 9.1 × 10^5^ CFU/g (range: 0.00–7.9 × 10^9^ CFU/g), while median TBL of CSS+ wounds was 1.2 × 10^7^ CFU/g (range: 0.00–1.0 × 10^9^ CFU/g; *p* < 0.001).

Average age of participants was 60.2 years (Table 2). At enrollment, 70% of study wounds exceeded 3 months duration. Among wounds that were considered CSS+, there was a significantly higher proportion of wounds prescribed antimicrobials (AM+) than those not prescribed antimicrobials (AM−, *p* < 0.001). Among wounds that were CSS−, there was a significant difference in the frequency of wound types among the AM+ and AM− subgroups (*p* = 0.01).

**Table 2.** Participant demographics. Values represent number of patients. Categorical data analyzed by chi-square test with p < 0.05 indicating statistical significance

		CSS+		CSS−			
	All Participants	+AM	−AM	Chi-Square Test	+AM	−AM	Chi-Square Test
Total *(n)*	350	36	12	*p* < 0.0001	220	82	*p* < 0.0001
Average age	60.2	57.1	59.7		60.3	61.4	
Gender							
Female	125	10	4		86	25	
Male	225	26	8		134	57	
Wound types							*p* = 0.01
DFU	138	18-	8		76	36	
PU	22	2	0		10	10	
SSI	60	2	1		40	17	
VLU	106	13	3		76	14	
Other	24	1	0		18	5	
Wound duration							
<3 months	106	12	2		72	20	
3–6 months	62	4	1		41	16	
6–12 months	56	6	1		35	14	
12+ months	126	14	8		72	32	
Prior systemic antibiotics^†^				*p* = 0.01			*p* < 0.0001
Yes	90	13	0		75	2	
No	260	23	12		145	80	

† On systemic antibiotic at time of study enrollment. Statistical significance indicated by *p*-values, in all other comparisons, no statistical significance was observed.

+AM, prescribed antimicrobials (including dressings and topicals, topical antibiotics, and systemic antibiotics); −AM, no antimicrobials prescribed; CSS+, three or more clinical signs and symptoms of infection detected; DFU, diabetic foot ulcer; PU, pressure ulcer; SSI, surgical site infection; VLU, venous leg ulcer.

Baseline systemic antibiotic use was significantly different within CSS+ cases and CSS− cases; these differences remained significant after adjusting for multiple comparisons.^16,26^ After correcting for multiple comparisons, odds of prescribing antimicrobials were 2.8-fold greater for those patients with a venous leg ulcer compared with those without (*p* = 0.0001). No other demographic variable was significantly associated with antimicrobial prescribing.

**Figure 2.**
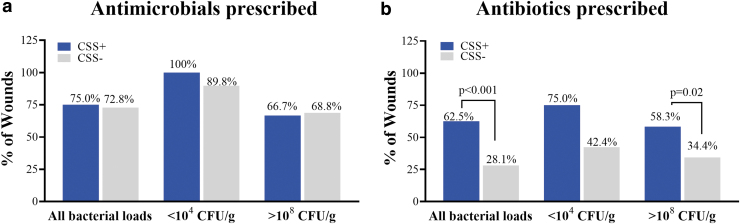
Antimicrobial **(a)** and antibiotic **(b)** prescription based on assessment of CSS, patient history, and clinical judgment. Antimicrobials included topicals, dressings and antibiotics (topical or systemic), antibiotics included topicals, oral or intravenous. Percentages reflect proportion of CSS+ and CSS− wounds among all participants (*n* = 350), participants with bacterial loads of <10^4^ CFU/g (*n* = 63), and participants with >10^8^ CFU/g (*n* = 44). *p* values derived from chi-square tests after correcting for multiple comparisons. Any comparisons for which *p*-values are not shown were not significant.

Antimicrobials (including bandages, topicals, or oral, topical or intravenous antibiotics) were prescribed in 73.1% (256/350) of treatment plans based on CSS assessment. Antimicrobials of any level were prescribed to a similar proportion of CSS+ (75.0% of CSS+ wounds) and CSS− wounds (72.8% of CSS− wounds; chi-squared = 0.097; *p* = 0.75, Fig. 2a). However, due to the larger proportion of study wounds identified as CSS− (302 wounds in total), antimicrobial prescriptions were 6.9-fold higher for CSS− wounds (*n* = 220) compared with CSS+ wounds (*n* = 36).

To evaluate whether antimicrobial and antibiotic prescribing rates correlated with bacterial burden, we analyzed antimicrobial and antibiotic prescribing trends in wounds with bacterial loads <10^4^ CFU/g (considered low risk of developing complications related to infection) and wounds with >10^8^ CFU/g, in which risk of developing infection-related complications is higher. Clinicians used CSS evaluation to infer presence of bacterial burden in wounds and did not have access to microbiological results at time of treatment planning. All CSS+ wounds with <10^4^ CFU/g were prescribed antimicrobials, but only 66.7% of CSS+ wounds with >10^8^ CFU/g were prescribed antimicrobials.

Interestingly, although the presence or absence of CSS did not influence overall prescription rate of antimicrobials, it did influence the type of antibiotic used. Rate of antibiotic (topical or systemic) prescription was 2.2-fold higher in CSS+ wounds (62.5%) compared with CSS− wounds (28.1%, Fig. 2b; *p* < 0.001). Among CSS− wounds prescribed antibiotics, systemic antibiotics (oral or intravenous) were prescribed 91% of the time. Surprisingly, antibiotic prescribing rate was highest (75.0%) in CSS+ wounds with bacterial loads <10^4^ CFU/g.

**Figure 3.**
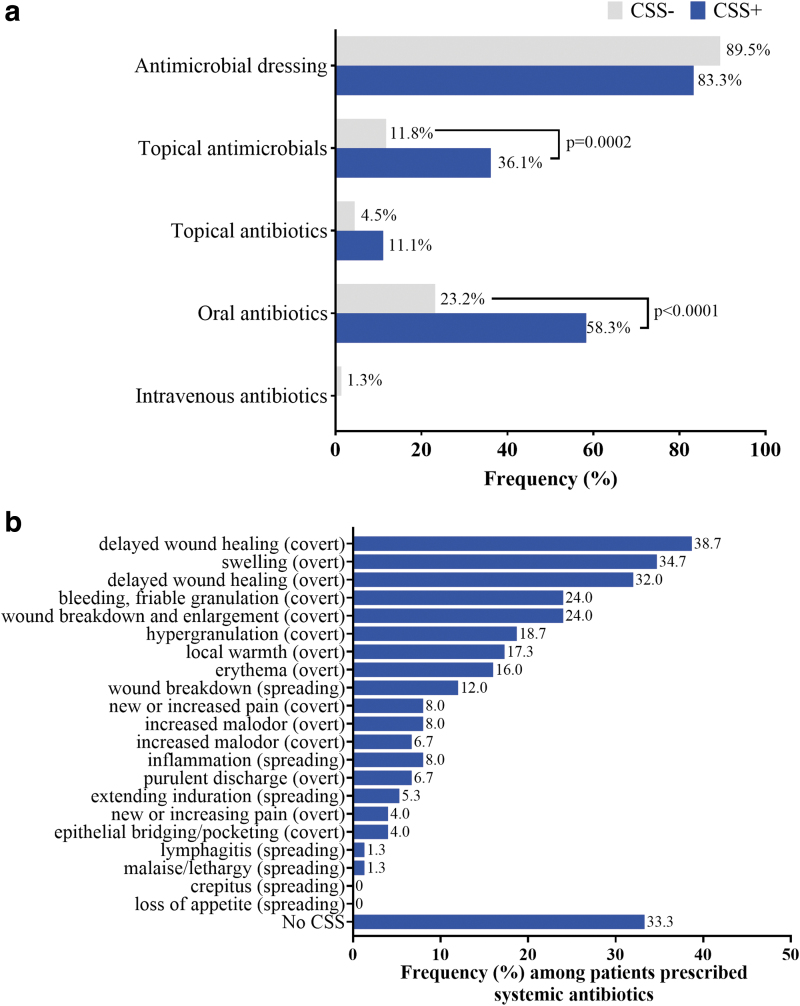
**(a)** Types of antimicrobials prescribed based on patient history and visual assessment of CSS of infection. Wounds with three or more CSS based on IWII criteria were considered positive for CSS (CSS+). Frequency represents the proportion out of the total number of antimicrobials prescribed for either CSS+ or CSS− wounds. **(b)** Frequency of specific CSS detected in patients prescribed systemic (oral or intravenous) antibiotics. Values represent % of all patients prescribed systemic antibiotics. Covert, overt, and spreading represent the designated categories of CSS in the IWII guidelines. *p* values derived from chi-square tests after correcting for multiple comparisons.

Antimicrobial bandages were the most frequently prescribed antimicrobial, and this was consistent among wounds deemed CSS+ (89.5% of CSS+ wounds prescribed antimicrobials) and CSS− (83.3% of CSS− wounds prescribed antimicrobials; Fig. 3a). Antimicrobial topicals were more frequently prescribed for CSS+ wounds (36.1%) compared with CSS− wounds (11.8%; *p* = 0.0002).

In 23.2% of CSS− patients prescribed antimicrobials, oral antibiotics were prescribed; CSS+ wounds were 2.5 times more likely to be prescribed oral antibiotics (58.3%) compared with CSS− wounds (23.2%; *p* < 0.0001). Intravenous antibiotics were not included in the treatment plan for any CSS+ wounds but were included in the treatment plans of three CSS− wounds (1.3%). Doxycycline (28.3%) was the most common oral antibiotic prescribed, followed by Cephalexin (18.9%).

Common CSS detected among patients prescribed systemic antibiotics included delayed wound healing (covert, 38.7%), swelling (overt, 34.7%), delayed wound healing (overt, 32.0%), bleeding, friable granulation (covert, 24.0%), and wound breakdown and enlargement (covert, 24.0%; Fig. 3b). Systemic antibiotics were provided in the 2 weeks before the study in 52/53 CSS− patients prescribed systemic antibiotics based on CSS assessment. In 33.3% of patients prescribed systemic antibiotics, no CSS were detected.

## Discussion

Understanding current trends in antimicrobial prescribing from outpatient wound clinics is of significant interest as clinicians, policy makers, and researchers work to implement antimicrobial stewardship programs. Clinicians are taught to assess the wound for CSS—the host response to excessive bacterial burden—to determine if antimicrobials are warranted.^10,12^ This is the first study to examine the relationship between antimicrobial prescribing, CSS, and TBL.

Data presented here demonstrate the scope and gravity of the bacterial burden problem in chronic wounds. Pathogenic bacterial loads^27^ were highly prevalent (>80% of wounds) but went largely undetected based on clinical assessment.^23^ The inability of standard-of-care assessment to detect wounds with significant bacteria burden undoubtedly contributed to inconsistent and haphazard antimicrobial prescribing practices for chronic wounds.

Perhaps due to the inherent uncertainty of CSS, clinicians in this study prescribed antimicrobials regardless of whether CSS were absent or observed. Consistent with prior studies,^18,19,28^ CSS was a poor indicator of concerning levels of bacteria in wounds. Almost half of the wounds judged by clinicians to lack CSS harbored >10^6^ CFU/g, loads that have been associated with infection.^17,29,30^

Antibiotic prescription patterns did not correlate with bacterial load, nor to the presence of CSS, resulting in prescribing practices that ranged from inconsistent to alarming. Over and underprescribing was rampant. Prescribing trends observed included a higher rate of antimicrobials prescribed for wounds that appeared not to require it (*e.g.,* administration of IV antibiotics for three CSS− wounds), as well as underprescribing for wounds with the highest bacterial loads (only 66.7% of CSS+ wounds with clinically significant loads of >10^8^ CFU/g received any level of antimicrobial).

These baffling prescribing practices, observed across multiple wound care centers, demonstrate how current empirical practices propagate antibiotic abuse. They also show how poor clinical decision making at the individual level contributes to the global and pervasive problem of antibiotic resistance. Antimicrobial stewardship efforts cannot succeed if these empirical practices are not reformed.

Key challenges in practically applying guidelines on antimicrobial use into clinical practice included the following:

(1) Underappreciation of the prevalence and severity of the bacterial burden harbored by chronic wounds. Findings from this study and the FLAAG trial indicate that most chronic wounds (>80%) treated in outpatient wound care centers have clinically significant levels of bacterial burden (>10^4^ CFU/g). However, these wounds remain undetected due to lack of accurate point-of-care methods to identify bacterial burden in wounds, contributing to delayed healing.
(2) Lack of universally accepted standards for diagnosing infection in chronic wounds. Assessment of CSS has been used since the Egyptian era to indicate infection and guide treatment selection,^31^ yet there is a lack of universally accepted criteria of infection.^10,12^ Although numerous checklists and guidelines have been developed to aid wound care providers in identifying CSS,^24,32^ there is considerable variation in these criteria across publications. In addition, the sensitivity and validity of these criteria for identifying infection are poor.^17,19^ As such, their utility in informing antimicrobial decision making at the point of care is questionable.
(3) Lack of real-time information on bacterial burden in wounds. If antimicrobial therapy is required, clinicians are advised to incorporate microbiological culture results (i.e., bacterial load, speciation, and antibiotic resistance) into their prescribing decisions. However, these results often take days or weeks to obtain. Further, recent evidence calls into question the reliability and utility of semiquantitative culture to determine bacterial quantity.^25^

Due to these challenges, clinicians have come to rely heavily on empirical evidence to guide antimicrobial prescription decisions. Until more reliable and objective methods to diagnose bacterial burden in wounds become widely used, antimicrobial prescribing will continue to be reliant on the subjectivity of CSS.

Underlying comorbidities may make infection challenging to diagnose in wounds if CSS fail to mount or are mimicked by other conditions.^12^ Faced with this uncertainty, physicians may prefer to overtreat bacterial burden rather than miss an infection, with the notion “It doesn't hurt, and it may help.” This preference is likely fuelled by fear that elevated bacterial loads that are undiagnosed may result in delayed wound healing and could lead to more invasive infection or other costly and serious consequences such as hospitalization and amputation.^32,33^

Indeed, clinically infected DFUs will almost always require antimicrobial therapy.^33^ In addition, fear of litigation exists if a patient develops a complication from a wound infection and antimicrobials were not used.^34^ As a result, the threshold to prescribe antimicrobials is low. In one retrospective study of children with uncomplicated skin and soft tissue infections, avoidable antibiotic exposure occurred in approximately half of infections.^35^

Overtreatment with systemic antibiotics in chronic wound patients who have multiple comorbid illnesses increases the risk of systemic complications, including renal failure, allergic reactions, drug interactions, and *C. difficile* colitis.^36^ The results of overprescribing in the outpatient setting contribute to emergence of multidrug-resistant bacteria and lead to poor quality clinical outcomes.^37^

The use of multidisciplinary antimicrobial stewardship teams and point-of-care diagnostics that provide objective information on bacterial burden should be considered to improve trends in antimicrobial prescribing strategies. Wound care best practice guidelines stress the importance of multidirectional flow of accurate and meaningful information within the entire wound care team.^22,24^ An ASP leader can reinforce the stewardship principles throughout the wound care center supporting the Joint Commission mandate.^22^

Although ASP teams are more common in hospital inpatient settings,^12^ others have advocated for more widespread implementation of such interdisciplinary ASP teams.^12,22^ The alarming antimicrobial prescribing trends in outpatient wound care centers reported here suggest that implementation of ASP teams beyond the inpatient setting is warranted. To assemble an ASP requires coordination and adoption across an institution that may make this a long-term goal. In the short term, there are strategies that can be implemented immediately to enhance antimicrobial prescribing, including greater emphasis on evidence-based decision making.

Assessment of CSS may initiate a clinical decision-making workflow that also includes more objective, diagnostic tests to detect bacterial burden at the point-of-care and support more thorough wound hygiene strategies before deciding to prescribe antimicrobials. There are several point-of-care diagnostics that have emerged to enhance detection of bacterial burden or infection in wounds. These include a point-of-care test to detect elevated protease activity,^38^ a wound dressing that changes color to indicate presence of pathogenic organisms,^39^ and a noncontact imaging device that enables visualization of fluorescence from wound bacteria at loads >10^4^ CFU/g at the patient bedside.^14,40,41^

Prior studies suggest that objective diagnostic imaging of bacterial loads can reduce the uncertainty of diagnosis and support more judicious use of antimicrobials, including antibiotics.^14,41^ Until more objective information on bacterial burden is implemented into our routine practices, antimicrobial resistance will continue to be a pervasive problem in wound care.

### Limitations

Data analyzed in this study was from a single visit trial that captured antimicrobial/antibiotic prescribing at one time point for each subject; as such, there was a lack of follow-up to correlate the choice of antimicrobial treatment to wound outcome. The diversity of the wound types and durations in this study is both a strength and a weakness. Although the wound types included are reflective of those typically treated in outpatient wound centers, there were additional factors (*e.g.*, wound clinician background, training) that may influence antimicrobial prescribing.

In addition, the decision to prescribe antimicrobials may be an artifact of the health system environment. In the United States, fear of potential litigation is a major determining factor in ordering medications, resulting in the practice of “defensive medicine.”^42^ Potential influence of this on antimicrobial prescribing was not examined herein; replication of this study in other jurisdictions is warranted.

**Key Findings**

- The scope of bacterial burden in wounds has been underestimated; 52.2% of study wounds had bacterial loads >10^6^ CFU/g, and 80.2% of wounds deemed by clinicians to lack CSS of infection had bacterial loads that can impair healing (>10^4^ CFU/g).
- The current reliance on clinical assessment for antimicrobial prescribing practices results in haphazard prescribing. Antimicrobials were prescribed at a similar rate for wounds deemed to be positive (CSS+, 75.0%) or negative (CSS−, 72.8%) for signs and symptoms of infection.
- Antimicrobial prescribing did not correlate with bacterial loads in chronic wounds.
- Until better methods for bacterial assessment are implemented, we cannot expect more prudent antimicrobial usage and success of antimicrobial stewardship programs.

## About the Authors

**Thomas E. Serena, MD, FACS, FACHM, FAPWCA** is Founder and Medical director of the SerenaGroup, a family of wound, hyperbaric, and research companies and is board certified in Surgery with practice in wound healing.

**Lisa Gould, MD, PhD, FACS** is the Director of Research at South Shore Hospital Center for Wound Healing, Weymouth, MA. She has served on the executive board of the Wound Healing Society for >10 years.

**Karen Ousey, PhD, RGN, FHEA, CMgr MCMI** is Professor of Skin Integrity and Director for the Institute of Skin Integrity and Infection Prevention at the University of Huddersfield.

**Robert S. Kirsner, MD, PhD** is Professor of Dermatology in the Dr. Phillip Frost Department of Dermatology and Cutaneous Surgery at the University of Miami Miller School of Medicine. He is also Director of the University of Miami Hospital and Clinics Wound Center.

## Abbreviations and Acronyms

AM: antimicrobials (including dressings, topical antimicrobials and topical, oral or intravenous antibiotics)
ASP: antimicrobial stewardship plan
CFU: colony forming units
CSS: clinical signs and symptoms
DFU: diabetic foot ulcer
FL: fluorescence imaging
FLAAG: fluorescence imaging assessment and guidance
IWII: International Wound Infection Institute
TBL: total bacterial load
VLU: venous leg ulcer
